# Supraparticles with silica protection for redispersible, calcined nanoparticles†

**DOI:** 10.1039/c9na00442d

**Published:** 2019-09-27

**Authors:** Susanne Wintzheimer, Franziska Miller, Johannes Prieschl, Marion Retter, Karl Mandel

**Affiliations:** University Würzburg, Chair of Chemical Technology of Materials Synthesis Röntgenring 11 97070 Würzburg Germany karl-sebastian.mandel@isc.fraunhofer.de; Translational Center Regenerative Therapies, TLZ-RT, Fraunhofer Institute for Silicate Research, ISC Neunerplatz 2 97082 Würzburg Germany; Fraunhofer Institute for Silicate Research, ISC Neunerplatz 2 97082 Würzburg Germany

## Abstract

Calcination of nanoparticles is always accompanied by undesired sintering. A calcination route preventing hard-agglomeration to bulk lumps, which is transferable to almost any kind of metal oxide nanoparticle, is developed by surrounding targeted nanoparticles by silica nanoparticles within a nanostructured microparticle. After calcination, the desired nanoparticles are regained as a monodisperse sol *via* silica dissolution.

## Abstract 2

The herein presented straight forward and upscalable silica-protected calcination route, using supraparticles as intermediary structures, is applicable to almost any kind of metal oxide NPs and prevents their hard-agglomeration to bulk lumps.

## Graphical abstract

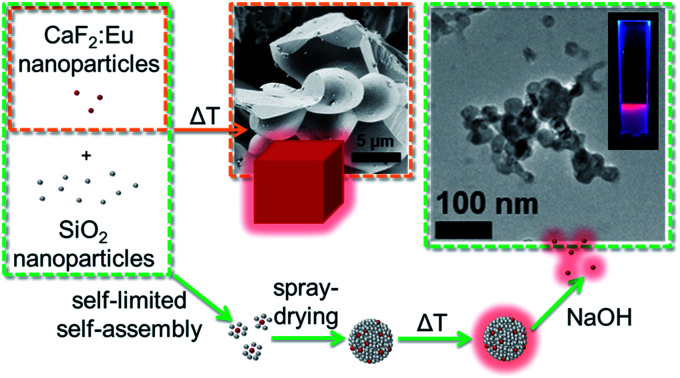

Calcination is a heat treatment step which is indispensable for the synthesis or to unfold the desired physical properties of a vast variety of nanoparticles (NPs) including hydroxyapatite ceramics,^1^ SnO_2_,^2^ CeO_2_,^3^ Cr_2_O_3_,^4^ TiO_2_,^5^ ZrO_2_,^6^ Co_3_O_4_,^7^ MgO^8^ and Y_2_O_3_.^9^ The calcination process is required to increase the amount of crystalline phase and to decrease crystal defects within the NPs.^2,6^ It can promote desired crystal phase conversions and remove unwanted impurities left from synthesis.^2,3,5^ Calcination of rare-earth containing crystals enhances their luminescence emission intensity due to a better crystallinity (*i.e.*, less defect states) of the host material, and a better positioning of the rare-earth ions within the crystal lattice.^6,9^

However, it is well-known that calcination of NPs always comes with the unwanted side-effect of sintering, *i.e.*, an increase of crystallite sizes and formation of hard-agglomerates.^2,3,5^ This development of solid bridges between particles is extremely disadvantageous as hard-agglomerates cannot be disintegrated by simple means but strong mechanical forces are required to break them down to smaller entities.^10^

In order to avoid sintering, NPs can be protected by a silica coating^11–15^ or encapsulation,^16^ which is applied *e.g. via* a Stöber process onto the particle surfaces.^17^ An alternative approach is to embed NPs in a silica-based hybrid gel.^18^ In all cases, the sinter-resistant silica encages the more temperature-labile core material thus preventing its sintering.^19^ This has already shown success in temperature treatments up to 800 °C in the case of Pt or Pd NPs used in catalyst applications.^11,14^ As a coating of the NPs can be disadvantageous for their later application, a few research groups have also shown that after heat treatment the silica coating can be removed *via* etching in a strong base.^20,21^

Another approach to protect NPs, such as FePt, against sintering is to intermix them with a much larger proportion of NaCl microparticles. Similar to a silica coating, the NaCl encloses the temperature-labile NPs as matrix, enabling their sinter-free annealing and their retrieval by dissolving NaCl in water.^22^

Based on these insights, in this current work presented herein a route toward the fabrication of calcined NPs was developed using supraparticles^23^ as intermediary structures. These micron-sized, hierarchical particles consist of silica NPs and another NP type (*e.g.* CaF_2_ or TiO_2_), which needs to be protected from sintering during calcination. They are assembled upon self-limited self-assembly^24,25^ (SLSA) combined with subsequent spray-drying.^26^ This procedure affords high flexibility in terms of building block selection (comparable to a toolbox), *i.e.*, it is possible to readily select the sizes, shapes and material types of the primary particles. It consequently enables the straight forward protection of any desired NP with silica (or other sinter-resistant) NPs in a robust, continuous and upscalable process. This displays a significant advantage over approaches using silica coatings, which have to be developed from scratch for every single targeted NP type and are difficult to upscale. In detail, careful control of wet nanochemistry on a larger scale, *e.g.* aiming for the production of several hundred grams to kilograms of particles, is a true challenge. Nearly any synthesis protocol has to be modified and *e.g.* working with highly diluted dispersions, which becomes necessary to be able to control the process, renders it impractical and unfeasible.

As a more detailed outline of this issue would be beyond the scope of this article, the interested reader is referred to a detailed discussion on this issue, which was recently published by us.^27^

The obtained supraparticles resemble raspberries in their structure^28^ and allow the calcination of the desired NP type. After calcination of the supraparticles, the (non-silica) NPs are regained as monodisperse sol *via* dissolution of the silica components in a strong base. Fig. 1 depicts the principle of this fabrication route using silica NPs as protectant against sintering, which is similar to foam peanuts as protectant against mechanical damage in the packaging of fragile products. However, it should be noted here that a difference between using foam peanuts for transport (as well as the mentioned approaches of embedding NPs in a gel or a salt matrix) and the herein proposed method is the formation of supraparticles instead of simply mixing both nanoparticles types. This brings the advantage of providing a free flowing micropowder sample after calcination (Fig. S1 in the ESI†) and thus, faster dissolution of the silica components due to a better accessibility compared to bulk material.

**Fig. 1:**
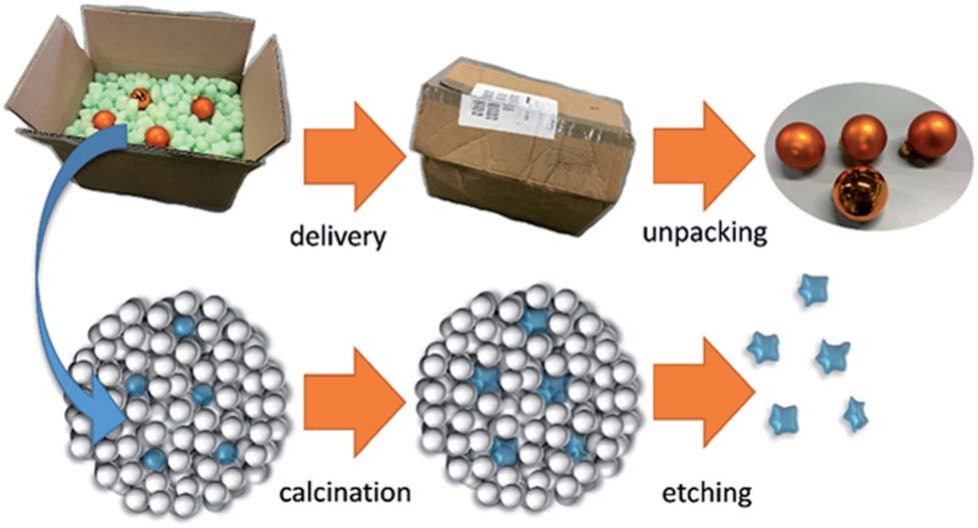
Similar to foam peanuts, which are used as protectant against mechanical damage in the packaging of fragile products, SiO_2_ NPs protect sinter-labile NPs within a supraparticle during calcination.

In order to optimally protect NPs by silica within a supraparticle during calcination, the spatial separation of sinter-labile NPs from each other is a crucial parameter. This inner structural arrangement of a supraparticle during the kinetic trapping is affected by the number ratio and size ratio of silica to NPs that need to be protected, as well as by the differences in surface charge of both NP types. Several publications have already studied supraparticles made of binary NP dispersions by means of spray-drying assisted assembly.^26,29–31^ Based on the findings of these studies, the conclusion can be drawn that for an efficient separation of one particle type by silica, not only silica NPs have to prevail in excess, but also both NP types need to be similar in size (Fig. S2a in the ESI†). This is at least the case for equally charged NPs displaying high surface charges and a good dispersion state in water before spray-drying^29^ (Fig. S2b, case I. in the ESI†). However, comparing the surface charge, *i.e.*, the zeta potential of silica with the one of other metal oxide NPs, it appears that the metal oxide NPs usually have their isoelectric points at higher pH values than silica (shown for the samples studied in this work in Fig. S3 in the ESI†).^32^

Thus, obtaining oppositely charged binary dispersions at pH values lying between the isoelectric points of both materials is most practicable in the majority of cases. This is why the concept of so-called SLSA^24^ of binary oppositely charged NP dispersions (Fig. S2b, case II.†) was connected upstream of the forced microparticle assembly *via* spray-drying. If the negatively charged silica NPs self-assemble around the sinter-labile NP type, the chance of an ideal separation of these NPs from each other within the supraparticle is increased. Furthermore, differences in NP size should no longer induce a segregation of the two NP types within the spray-dried supraparticles. Thus, an efficient separation of the sinter-labile NPs is guaranteed as long as the silica displays the higher particle number and the smaller particle size in order to form the outer layer during the SLSA^33^ (which is shown for CaF_2_ and two sizes of silica NPs in varying weight ratios in Fig. S4 and S5 in the ESI†). The process flowsheet of the continuous double assembly of Eu^3+^ containing CaF_2_ as exemplary sinter-labile NPs with silica NPs is shown in Fig. 2a (and Experimental details can be found in the ESI†). The aqueous silica and CaF_2_ sols are pumped into a static mixer in a NP weight ratio of 9 : 1. This results in a dispersion of well-separated silica NPs of around 20 to 30 nm in size and soft-agglomerates of CaF_2_ NPs of around 200 nm (Fig. 2b1 and b2). Ultrasound application enables the SLSA of silica NPs around the CaF_2_ NPs yielding soft-agglomerates of around 100 nm in size besides well-separated silica NPs due to their number surplus (Fig. 2c1 and c2). Subsequent spray-drying provides nanostructured microparticles consisting of CaF_2_ NPs embedded into silica NPs (Fig. 2d). This supraparticle structure enables the calcination without hard-agglomeration to bulk lumps up to at least 800 °C (Fig. S6 in the ESI† in comparison to spray-dried CaF_2_ NPs without silica protection).

**Fig. 2:**
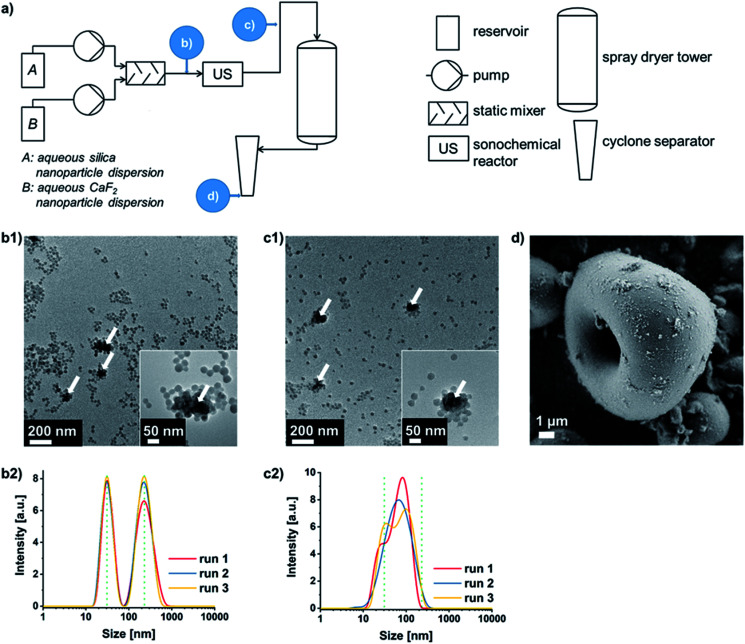
(a) Schematic process flowsheet of the continuous double assembly process with analyses carried out at different time points (b–d): transmission electron microscopy (TEM) images (b1 and c1, white arrows indicating CaF_2_ NPs) and intensity weighted agglomerate sizes determined *via* dynamic light scattering (DLS, b2 and c2) before and after ultrasound-assisted SLSA of the binary NP dispersion, respectively. (d) Scanning electron microscopy after forced assembly of the pre-assembled binary NP dispersion by spray-drying.

In order to highlight the benefits of the silica-protected calcination route, the properties of the obtained Eu^3+^ containing CaF_2_ NPs after calcination at 600 °C were compared to the as synthesized CaF_2_ crystals (Fig. 3a and b). While the rare-earth luminescence is significantly enhanced by the calcination, the crystallite size calculated from X-ray diffraction (XRD), using the Scherrer equation, is only slightly increased from 21 ± 3 nm to 26 ± 1 nm. TEM and DLS measurements also confirm the nanoscale sizes of the single-crystalline particle samples. While the measured NP size on TEM micrographs increases from 28 to 46 nm (Fig. S7 in the ESI†), the agglomerate size around 100 nm obtained from DLS shows the dispersion state of the samples in water and indicates soft-agglomeration of the nanoparticles. This soft-agglomeration also complicates the particle size determination *via* TEM. The increase in size shown by TEM and XRD indicates a slight sintering due to coalescence. However, the nanoscale size and nano-redispersibility of the sample are maintained.

**Fig. 3:**
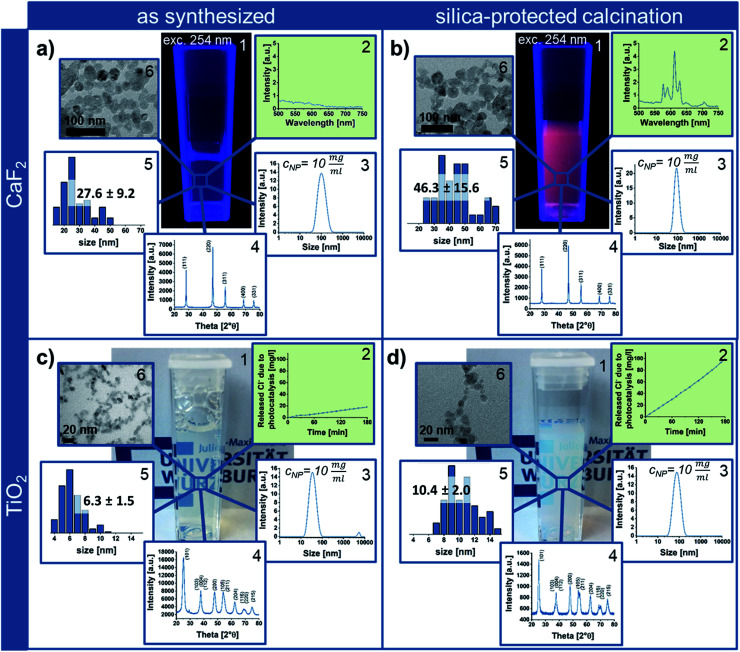
Eu^3+^ containing CaF_2_ (a and b) and anatase NPs (c and d) in the as synthesized state (a and c) compared to calcined samples obtained after silica-protected calcination (which also includes the removal of silica *via* etching and subsequent purification) for 4 h at 600 °C or 800 °C (b and d), respectively. (a) and (b) showing photographs (with UV-light irradiation) (1), photoluminescence spectra (2), intensity weighted agglomerate sizes determined *via* DLS (3; with *c*_NP_ indicating the sample concentration for measurements), XRD patterns (4) and nanoparticle size and size distribution obtained from TEM images measuring at least 50 particles per sample (5) as well as TEM images (6). (c) and (d) depicts the same analyses, having replaced the photoluminescence spectra by photocatalytic activity tests (2). The green background of the diagrams (2) highlights the improvement of NP properties by silica-protected calcination.

*Via* transfer of the silica-protected calcination route to anatase NPs (Fig. 3c) by adaptation of the silica NP size and amount, the easy portability of this process to other NP materials could be shown. Similar to the calcined silica-protected CaF_2_ NPs, the obtained single-crystalline TiO_2_ sample displays nanoparticulate size; the crystallite and TEM size only slightly increase from 6 ± 1 nm to 7 ± 1 nm and from 6 to 10 nm, respectively (Fig. 3d, while calcination without silica protection results in bulk material – Fig. S7 in the ESI†). However, the photocatalytic activity is significantly enhanced by the calcination process.

## Conclusions

In summary, a silica-protected calcination route for NPs, which prevents their hard-agglomeration to bulk lumps, using supraparticles as intermediary structures was herein presented. This procedure is straight forward, upscalable and readily transferable to almost any kind of metal oxide NP system yielding a significant improvement of properties while maintaining their nanoscale size.

## Conflicts of interest

There are no conflicts to declare.

## Supplementary Material

NA-001-C9NA00442D-s001
