# Supplementary material for: Supraparticles with silica protection for redispersible, calcined nanoparticles
Source: Nanoscale Adv. 2019 Sep 27;1(11):4277–81. doi: 10.1039/c9na00442d (PMC9417870; doi:10.1039/c9na00442d)
Supplement: NA-001-C9NA00442D-s001 [file NA-001-C9NA00442D-s001.pdf]

Electronic Supplementary Material (ESI) for Nanoscale Advances.
This journal is © The Royal Society of Chemistry 2019

Supporting Information

Supraparticles with Silica Protection for Redispersible, Calcined Nanoparticles

*Susanne Wintzheimer,[a] Franziska Miller,[a] Johannes Prieschl,[a] Marion Retter,[b] Karl Mandel[a,b,]**

[a] University Würzburg, Chair of Chemical Technology of Materials Synthesis, Röntgenring 11, 97070 Würzburg, Germany.

[b] Fraunhofer Institute for Silicate Research, ISC, Neunerplatz 2, 97082 Würzburg, Germany.

* corresponding author: karl-sebastian.mandel@isc.fraunhofer.de (K.M.)

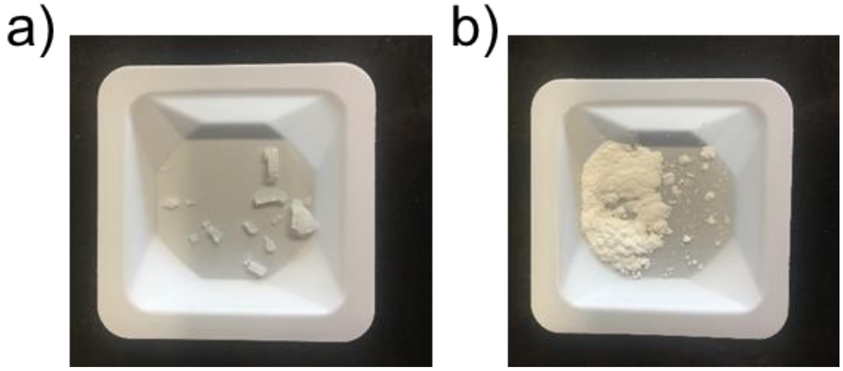

Figure S1. The simple mixing of CaF_2 and silica nanoparticle dispersions in a particle weight ratio of 1 : 9 and subsequent calcination at 600 °C for 4 h yields bulk material (photograph a), while an upstream supraparticle formation results in a free-flowing powder after calcination (photograph b).

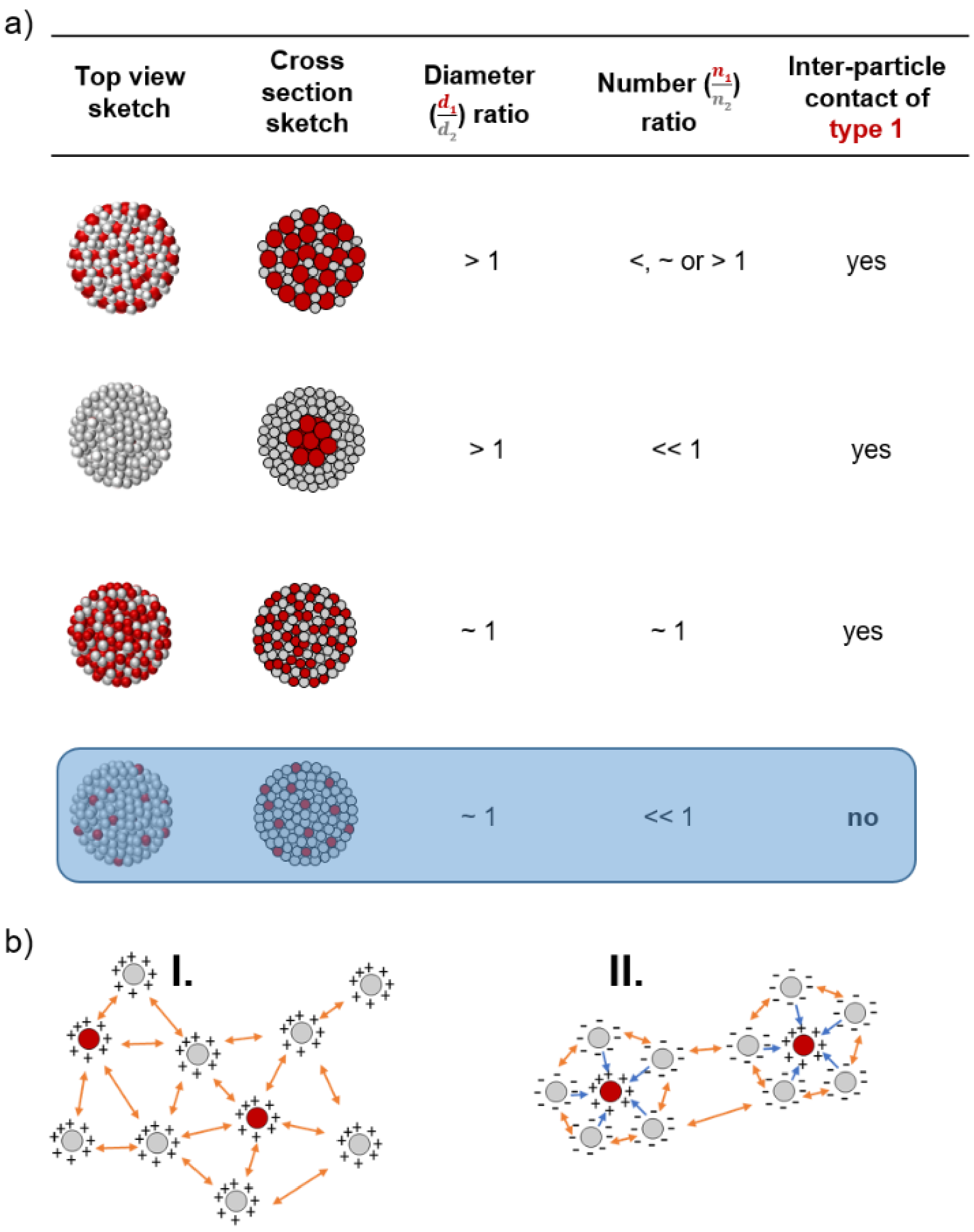

Figure S2. a) The influence of nanoparticle diameter and number ratio on the inner structure of binary spray-dried supraparticles and the inter-particle contact of one particle type (based on [1–4]). b) The electrostatic stabilization of binary nanoparticle dispersions in the case of equally charged (case I.) and oppositely charged (case II.) particle samples.

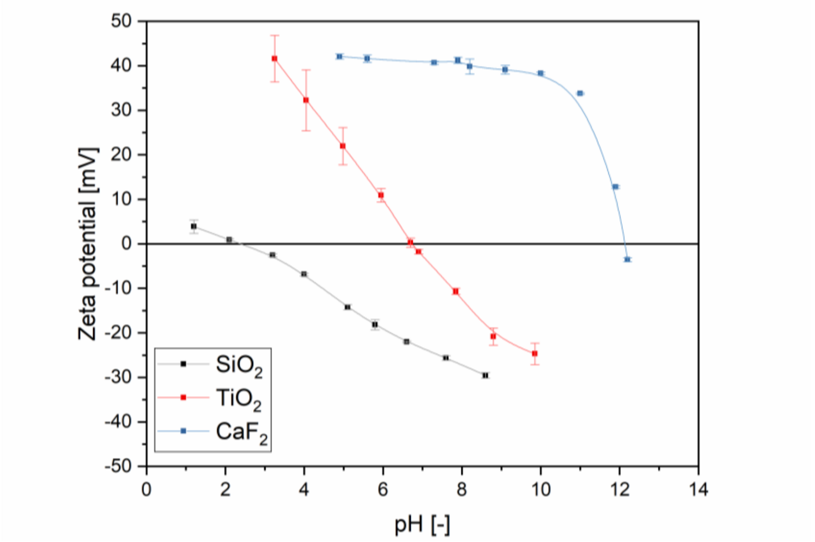

Figure S3. Zeta potentials over the pH of silica, CaF_2 and TiO_2 nanoparticle dispersions in water.

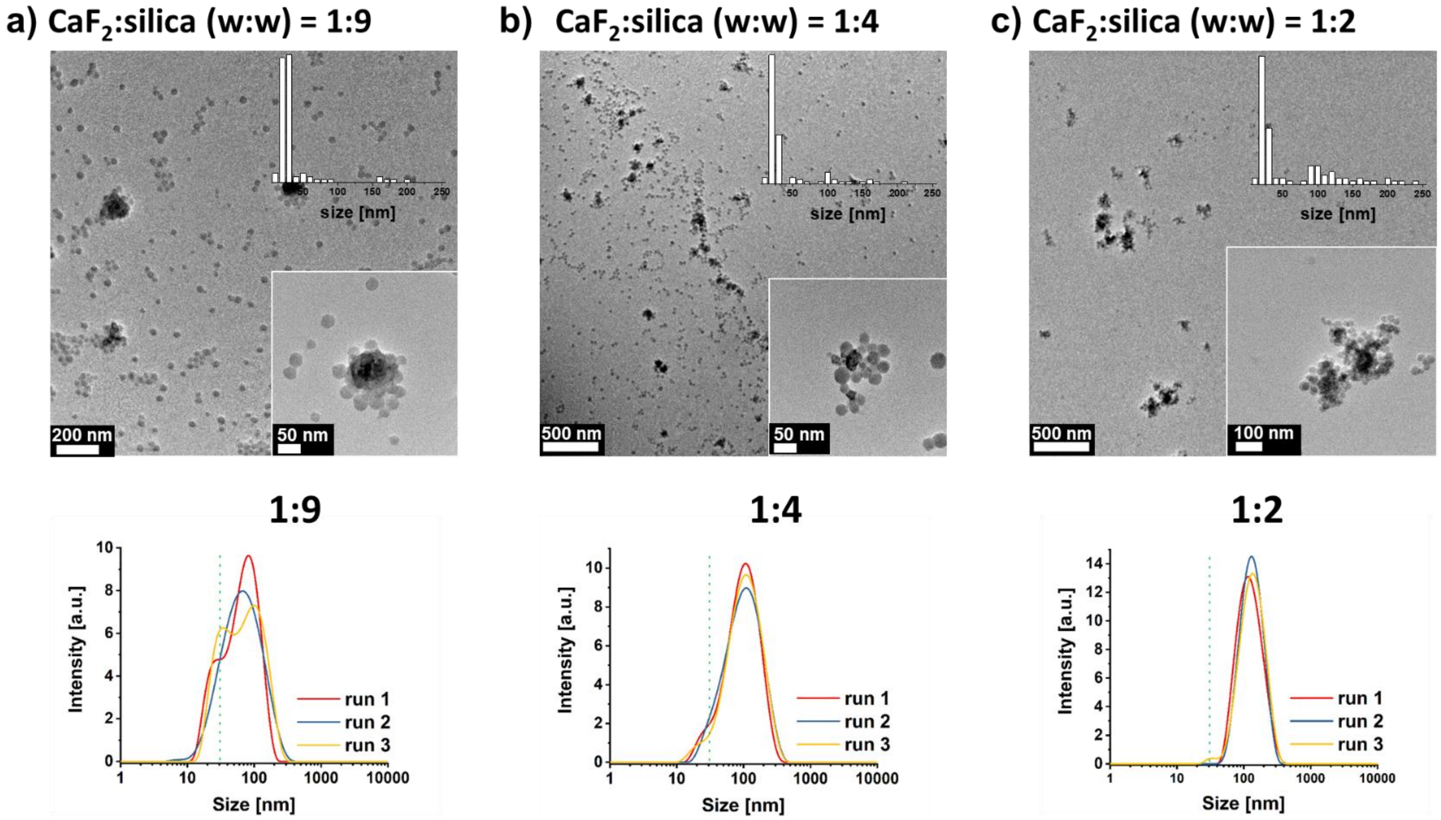

Figure S4. Transmission electron microscopy images with the nanoparticle size distribution obtained by measuring at least 100 particles per sample as insets (top row) and intensity weighted agglomerate sizes determined via dynamic light scattering (bottom row) of binary (CaF_2 and SiO_2) nanoparticle dispersions after ultrasound-assisted self-limited self-assembly. The nanoparticle weight ratios CaF_2 to SiO_2 vary from a) 1:9 to b) 1:4 and c) 1:2.

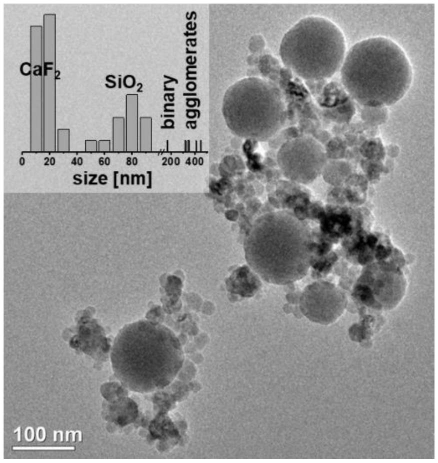

Figure S5. Transmission electron microscopy image with the nanoparticle size distribution obtained by measuring 100 particles (as inset) of binary (CaF_2 and SiO_2) nanoparticle dispersions after ultrasound-assisted self-limited self-assembly. The nanoparticle weight ratio of CaF_2 to SiO_2 is 1:9 and the silica particle diameter is around 95 nm. This consequently leads to soft-agglomerates with a silica core surrounded by CaF_2 nanoparticles.

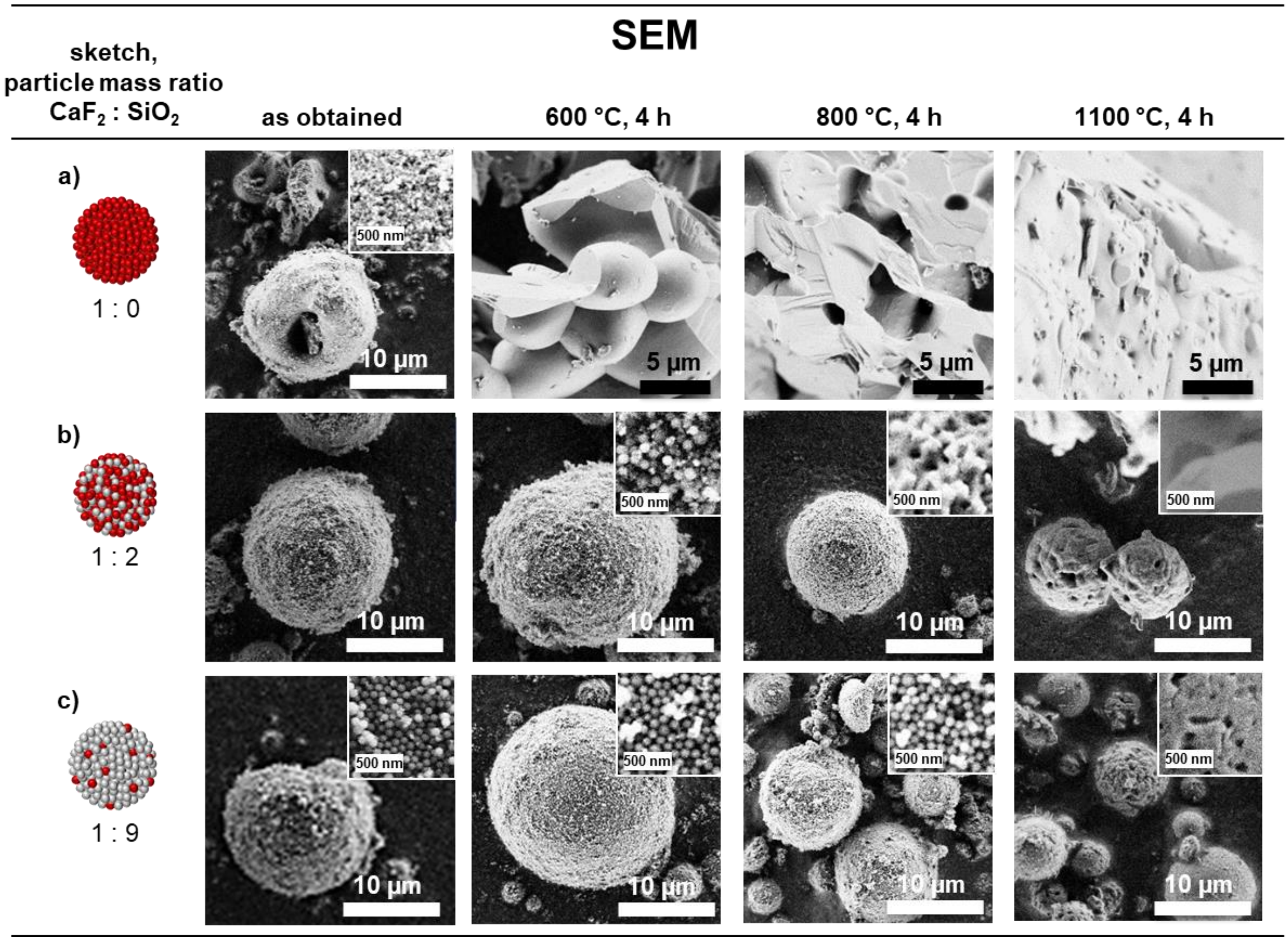

Figure S6. Scanning electron microscopy (SEM) images of spray-dried supraparticles as obtained and calcined for 4 h at 600, 800 or 1000 °C consisting of CaF_2 and SiO_2 nanoparticles with weight ratios of a) 1:0, b) 1:2 and c) 1:9. With increasing amount of SiO_2 nanoparticles within the supraparticles, the sintering of the supraparticles is more and more inhibited.

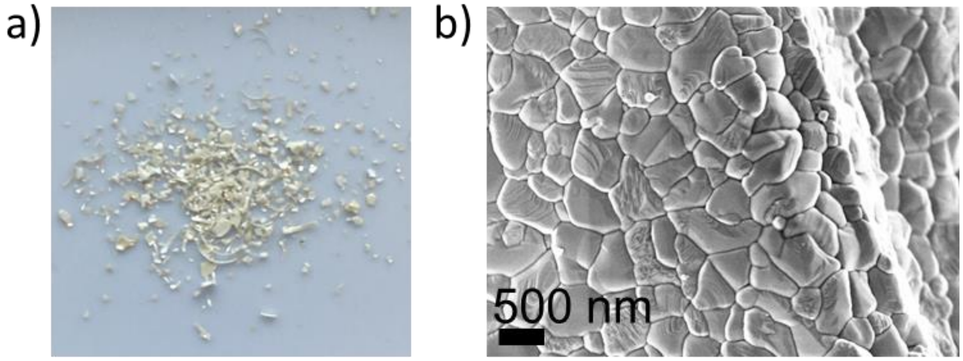

Figure S7. Photograph (a) and SEM image (b) of TiO_2 nanoparticles calcined for 4 h at 800 °C without any silica protection yielding bulk material.

Materials and Methods

Materials

Acetylacetone and p-toluene sulfonic acid monohydrate (98%) were purchased from Sigma–Aldrich. Calcium chloride and ammonium fluoride were provided by Merck. Titanium (IV) ethoxide was obtained from Lehmann & Voss & Co and europium (III) chloride hexahydrate from Easchem. Silica nanoparticles were used as an aqueous nanoparticle dispersion (Köstrosol 0830 AS and 2040 AS, Chemiewerke Bad Köstriz, Germany), containing 30 and 40 wt% silica with a diameter of about 8 and 20 nm, respectively. All chemicals were used without further purification. Water was deionized before use.

Synthesis of CaF_2 nanoparticles

CaF_2 nanoparticles were synthesized via a precipitation method;[5] Calcium chloride (13.84 g) and europium (III) chloride hexahydrate (0.46 g) were dissolved in water (120 ml). This solution was quickly added to ammonium fluoride (9.38 g) in water (120 ml) under stirring. The reaction mixture was stirred for 20 min. The precipitated CaF_2 nanoparticles were purified via repeated centrifugation and redispersion in water.

Synthesis of TiO_2 nanoparticles

TiO_2 NPs were synthesized via hydrothermal treatment[6]; Titanium (IV) ethoxide was mixed with acetylacetone as complexing agents and hydrolysed by dropwise addition of p-toluene sulfonic acid containing water over 1 h and stirring overnight at room temperature. The molar ratio of the educts was 1.00/ 0.67/ 3.33/ 0.03 (titanium (IV)

ethoxide/ acetylacetone/ water/ p-toluene sulfonic acid). After hydrolysis, all volatile constituents were removed via rotary evaporation. The obtained powder was dissolved in water to obtain a 12 wt-% amorphous titanium sol, which was subsequently hydrothermally treated in an autoclave (with 250 ml Teflon insert filled to 75 % volume) for 4 h at 160 °C. The resultant gels were dissolved in water (3 wt-%) and pressure filtrated (0.8 µm membrane).

Silica-protected calcination of CaF_2 nanoparticles

The double assembly of supraparticles was carried out as described in Figure 2a). For this, 150 ml water containing 3 g well-dispersed CaF_2 nanoparticles and 150 ml water containing 27 g well-dispersed Köstrosol 2040 AS SiO_2 nanoparticles were added in reservoir A and B, respectively. The pump rate of the peristaltic pump was selected to be 5 $ml \cdot min^{-1}$. The ultrasonic treatment was carried out at 180 W and the pH-value was set to be 8 during self-limited self-assembly. The final spray-drying was carried out on a ‘‘B-290” mini spray-dryer from Büchi (Switzerland). The inlet air temperature was chosen to be 130 °C and the outlet temperature 70–80 °C during the spray-drying process. After spray-drying, the produced supraparticulate powder was calcined in an oven at 600 °C for 4 h in air. For the silica dissolution, the calcined sample (3 g) was added to a 5 molar NaOH solution (27 g) stirred at 80 °C for 6 h and at room temperature for further 12 h. The regained CaF_2 nanoparticles were purified via repeated centrifugation and redispersion in water.

Silica-protected calcination of TiO_2 nanoparticles

The procedure was carried out as described for CaF_2 nanoparticles but reservoir A was filled with 25 ml water containing 0.5 g well-dispersed TiO_2 nanoparticles and B with 25 ml water containing 4.5 g well-dispersed Köstrosol 0830 AS. The pH-value was set to be 5 during ultrasound-assisted self-limited self-assembly The calcination of the produced supraparticulate powder was calcined at 800 °C for 4 h in air.

Characterization

Transmission electron microscopy (TEM) with a HITACHI H7650 at an acceleration voltage of 100 kV was used to examine the nanoparticle morphology (with samples prepared on carbon-coated copper grids). For the

size determination of nanoparticles a JEOL JEM-2100(HR) at an acceleration voltage of 200 kV was used and at least 50 nanoparticles (on at least 3 different images, which were representative for the respective sample,) were measured. Scanning electron microscopy (SEM) was carried out on a Zeiss Supra 25 SEM at 3 keV.

Dynamic light scattering and zeta potential analysis were conducted on the Zetasizer Nano ZS (Malvern Instruments) in combination with the Multi-Purpose Titrator MPT-2. The isoelectric points were determined during titration with the help of HCl and NaOH. For dynamic light scattering measurements, the particle concentration was always set to 10 mg particle per 1 ml of water. For measuring binary CaF_2 and silica dispersions, the refractive index was set to 1.475 of silica, which nevertheless is very close to the refractive index of CaF_2 (1.433).

The as synthesized and calcined CaF_2 and TiO_2 nanoparticle samples were analyzed via XRD on a PANalytical Empyrean Series 2 employing Cu Kα radiation. The crystallite size of the CaF_2 NPs was determined based on Scherrer's formula for the peaks (111) at 28.2° and (220) at 46.6° 2θ from the XRD (taking the average of the sum of these two peaks). For the calculation of the crystallite sizes of the TiO_2 NPs the peaks (011) at 25.2° and (200) at 47.8° 2θ were used.

Photoluminescence analysis on CaF_2 powder samples was carried out on a Jasco FP-8600 NIR Spectrofluorometer excited at 254 nm. The photocatalytic activity of TiO_2 nanoparticles was assessed via measuring produced chloride ions during the dichloroacetic acid degradation under UV light application as already described before.[7] The only modification was the reduction of the reactor volume and thus, of the measured solution to 0.5 L (water, 100 mg/L NP and 1 g/L dichloroacetic acid).

References

(1) Zellmer, S.; Garnweitner, G.; Breinlinger, T.; Kraft, T.; Schilde, C. Hierarchical Structure Formation of Nanoparticulate Spray-Dried Composite Aggregates. *ACS nano* **2015**, *9*, 10749–10757, DOI: 10.1021/acsnano.5b05220.

(2) Nandiyanto, A. B. D.; Okuyama, K. Progress in Developing Spray-drying Methods for the Production of Controlled Morphology particles: From the nanometer to submicrometer size ranges. *Adv. Powder Technol.* **2011**, *22*, 1–19, DOI: 10.1016/j.apt.2010.09.011.

(3) Balgis, R.; Ernawati, L.; Ogi, T.; Okuyama, K.; Gradon, L. Controlled Surface Topography of Nanostructured Particles Prepared by Spray-drying Process. *AIChE J.* **2017**, *63*, 1503–1511, DOI: 10.1002/aic.15682.

(4) Iskandar, F.; Chang, H.; Okuyama, K. Preparation of Microencapsulated Powders by an Aerosol Spray Method and their Optical Properties. *Adv. Powder Technol.* **2003**, *14*, 349–367, DOI: 10.1163/15685520360685983.

(5) O'Dwyer, C.; James, H. J.; Cheu, B.; Jaque, F.; Han, T.P.J. An Optical Investigation of Nano-Crystalline CaF_2 Particles Doped with Nd^{3+} Ions. *Chem. Phys. Lett.* **2017**, *685*, 217–221, DOI: 10.1016/j.cplett.2017.07.067.

(6) Koch, S.; Kessler, M.; Mandel, K.; Dembski, S.; Heuzé, K.; Hackenberg, S. Polycarboxylate Ethers: The Key Towards Non-toxic TiO_2 Nanoparticle Stabilisation in Physiological Solutions. *Colloids Surf., B* **2016**, *143*, 7–14, DOI: 10.1016/j.colsurfb.2016.03.010.

(7) Wintzheimer, S.; Szczerba, W.; GuilhermeBuzanich, A.; Kashiwaya, S.; Klein, A.; Jaegermann, W.; Toupance, T.; Shmeliov, A.; Nicolosi, V.; Heuzé, K. *et al.* Discovering the Determining Parameters for the Photocatalytic Activity of TiO_2 Colloids Based on an Anomalous Dependence on the Specific Surface Area. *Part. Part. Syst. Charact.* **2018**, *35*, 1800216, DOI: 10.1002/ppsc.201800216.
